# An Early Model for Value and Sustainability in Health Information Exchanges: Qualitative Study

**DOI:** 10.2196/medinform.9299

**Published:** 2018-04-30

**Authors:** Sue S Feldman

**Affiliations:** ^1^ Department of Health Services Administration University of Alabama at Birmingham Birmingham, AL United States

**Keywords:** health information exchange, medical informatics, information systems, value proposition, health informatics

## Abstract

**Background:**

The primary value relative to health information exchange has been seen in terms of cost savings relative to laboratory and radiology testing, emergency department expenditures, and admissions. However, models are needed to statistically quantify value and sustainability and better understand the dependent and mediating factors that contribute to value and sustainability.

**Objective:**

The purpose of this study was to provide a basis for early model development for health information exchange value and sustainability.

**Methods:**

A qualitative study was conducted with 21 interviews of eHealth Exchange participants across 10 organizations. Using a grounded theory approach and 3.0 as a relative frequency threshold, 5 main categories and 16 subcategories emerged.

**Results:**

This study identifies 3 core current perceived value factors and 5 potential perceived value factors—how interviewees predict health information exchanges may evolve as there are more participants. These value factors were used as the foundation for early model development for sustainability of health information exchange.

**Conclusions:**

Using the value factors from the interviews, the study provides the basis for early model development for health information exchange value and sustainability. This basis includes factors from the research: fostering consumer engagement; establishing a provider directory; quantifying use, cost, and clinical outcomes; ensuring data integrity through patient matching; and increasing awareness, usefulness, interoperability, and sustainability of eHealth Exchange.

## Introduction

### Background

The last decade has been one of understanding the contribution of the health information exchange to health care’s Triple Aim: improved care, lowered costs, and increased patient satisfaction. To that end, eHealth Exchange (formerly Nationwide Health Information Network [NwHIN]) was established in 2009 as the nation’s mechanism of health information exchange. However, onboarding was slow, and the US government soon realized that internal electronic exchange within an organization was not enough. Motivated by incentive funding provided by the Health Information Technology for Economic and Clinical Health (HITECH) Act, many states or regions have health information exchanges (HIEs), and many electronic health record (EHR) vendors are capable of health information exchange with disparate organizations. For the purposes of this paper, HIE refers to a single organization or group of organizations facilitating the act of electronic health information exchange. Additionally, eHealth Exchange is a vehicle facilitating health information exchange for HIEs.

While no solution, including eHealth Exchange, will singlehandedly address every health information exchange scenario, eHealth Exchange, as our nation’s HIE, is an environment and a component toward the ability to exchange records with any provider, at any time, for any patient. Isolated use cases and studies have tried to quantify the economic value of health data exchange across an HIE in general [1,2] and eHealth Exchange more specifically [3,4], and some have reported cost savings in terms of laboratory and radiology testing, emergency department expenditures, and admissions [2,5-7], one of which claims, “little generalizable evidence currently exists regarding benefits attributable to HIE” [7]. Additionally, models that consider both current and perceived value are needed to help move away from isolated use case examples and statistically quantify value and sustainability. As shown in the literature, value is not a singular focus, and therefore a method and model of statistically quantifying value that considers multiple factors is important.

### The Sequoia Project

The Sequoia Project, which partially funded this study, is a nonprofit membership corporation whose goal is to improve the health and welfare of all Americans by supporting and advancing health data exchange that is trusted, scalable, and enhances quality of care and health outcomes by supporting comprehensive longitudinal health records. The Sequoia Project seeks to expand trusted, secure, and interoperable exchange of health information across the nation by fostering cross-industry collaboration and consensus agreement among public and private organizations who wish to function as interconnected networks. Current eHealth Exchange participation includes over 100 organizations, representing about 33% of all US hospitals, over 17,000 medical groups, over 8200 pharmacies, over 1000 dialysis centers, and over 100 million patients.

### Theoretical Orientation: Group Forming Networks

While over 100 organizations participate in eHealth Exchange, there are few regional clusters/networks within which medical information is able to be queried and retrieved. Having regional clusters/networks would facilitate the transportation of vital information needed to provide a comprehensive clinical picture, exponentially (according to the premise of group forming networks) increasing the value of eHealth Exchange to *all* organizations. The Healthcare Information and Management Systems Society (HIMSS) [8] suggests that more needs to be done to show the business value of health data exchange and suggests value in terms of creating a health care data economy whereby people are willing to pay for and sell data, stakeholders could control data and exchange with others, and the surrounding ecosystem includes measures of interoperability that are meaningful to patients and providers.

This value equation has been seen in other network of networks configurations, described as group forming networks, or Reed’s Law. Reed describes 3 types of networks: a one-to-many network, in which a central entity shares information with a large number of members (eg, through a Web portal); a one-to-one network, where single members are connected to other individuals to conduct a number of transactions (eg, email); and a flexible communication network, which renders it possible to connect not only pairs of participants but groups as well. Metcalfe’s Law has also been used to describe network value but does not account for the power of group connections, in this case HIE networks or groups [9].

Under Reed’s Law, value grows such that the whole network (eHealth Exchange) is greater than the sum of the individual participants or clusters/networks (statewide or vendor HIE networks) [10]. This environment exponentially increases the number of health data exchange transactions that can occur and broadens the geographical reach of the individual and collective networks, thereby providing more accurate, current, and comprehensive information at the point of care. Furthermore, the expansion of accountable care models and retail medical clinics (eg, CVS MinuteClinic or Walgreens Healthcare Clinic) present additional opportunities for onboarding to state or regional HIEs, thereby bringing additional groups to eHealth Exchange (assuming the state or regional HIEs are themselves onboarded to eHealth Exchange). The use of such a network of networks could aid widespread achievement of the Triple Aim, widespread use of health information exchange in general and eHealth Exchange in particular, and increase the value of individual and collective factors. The purpose of this study was to explore the various factors associated with real and perceived value to provide a basis for early model development for health information exchange value and sustainability.

At a high level, Reed’s Law suggests that all connections result in some degree of value. A white paper from Brookings Center for Technology Innovation [11] provides more detail on this with a model showing connections between patients, payers, medical data providers, and health care providers. For example, more data between patients and health care providers could result in better care, and better care could result in lower costs between patients and payers.

## Methods

The study design incorporated 21 semistructured 1-hour phone interviews and document analyses to understand the perceived current and potential value factors of eHealth Exchange participation. Each interview was recorded and transcribed. Transcriptions were imported into ATLAS.ti (ATLAS.ti Scientific Software Development GmbH) for data organization and analysis. The findings from the interviews were used to form the basis of an early model for health information exchange value and sustainability.

Interviewees were recruited by email invitation and were purposefully selected based on their participation in the decision-making process to onboard to eHealth Exchange. All interviewees were consented and their identity, location, organization, and role within the organization will be kept confidential; interviewees came from the hospital system (7), statewide HIE (2), regional HIE (1), vendor (2), and federal government (9) sectors.

The following is an example of selected interview questions (a full listing can be found in Multimedia Appendix 1):

- What technical advances will need to happen for more organizations to join eHealth Exchange?
- What technical issues need to be solved to impact sustainability?
- What are the current reasons for maintaining your participation in eHealth Exchange?
- What needs to happen for HIE to impact improved care delivery, reduce costs, etc?
- What public policies need to happen for HIE to be a standard of care?

Using a grounded theory inductive approach [12], 16 conceptual categories and 73 subcategories emerged with relative frequency (RF) counts ranging from 0.20 to 6.77. RF, in this case, is the proportion of responses (as in frequency of a response) in the particular category across all interviews divided by the number of interviewees. For example, if 10 interviewees talk about the number of records being exchanged using eHealth Exchange as having to do with use, the RF would be 3.00. Using 3.00 as a cutoff, 5 main categories and 16 subcategories are described in the findings.

## Results

### Overview

The findings of this qualitative study reveal that a majority of eHealth Exchange participants have onboarded since 2014, even though eHealth Exchange originated in 2009 (as NwHIN). Overall, interviewees demonstrated much confusion regarding vendor HIEs, regional HIEs, statewide HIEs, and eHealth Exchange. At times in the interview process, interviewees needed to be recentered that the interview was specific to eHealth Exchange and not about other HIEs such as those contained within vendor systems. When interviewees were asked about the alignment of policy to health data exchange initiatives, many commented that public policy and legislation need to catch up to the willingness of providers to exchange information and of consumers to have their information exchanged.

Using RF≥3.00 as a top-tier cutoff for data reporting, Table 1 and Table 2 show 5 conceptual categories and 16 subcategories. To readily show the issues of greatest importance, Table 1 is organized in descending order (RF=6.77 to RF=3.00). To correspond to the narrative detail in this section, Table 2 is organized with the subcategory data grouped by category.

### eHealth Exchange Concerns and Challenges

The primary concerns expressed by interviewees related to interoperability (RF=6.17), level of implementation (RF=4.60), and increasing statewide or regional HIE to eHealth Exchange connectivity (RF=3.17).

### Interoperability

When interviewees discussed reconciling technology and usability, they pointed out that eHealth Exchange is not plug and play and lamented the lack of direct communication from vendors about their system requirements. One interviewee summed up what many expressed: “Make it [eHealth Exchange] as interoperable as banking.” Even still, interoperability will require constant consensus building, improvement, and course corrections to keep pace with innovations.

**Table 1:** Overall findings of the interviews in descending order (relative frequency [RF] ≥3.00).

Category	Subcategory	RF
Value	Value in better care	6.77
Use	Increase eHealth Exchange use	6.75
eHealth Exchange concerns or challenges	Interoperability	6.17
Technical	Technical standards	6.13
Technical	Patient matching	5.80
Value	Value in avoiding duplication	4.93
eHealth Exchange concerns	Level of implementation	4.60
Value	Value in lowering costs	4.47
Technical	Data usability	4.37
Use	Who is using eHealth Exchange	4.30
Technical	Data integrity	3.87
Value	Intangible value	3.53
Use	Actual eHealth Exchange use time	3.30
eHealth Exchange concerns	Increase statewide health information exchange to eHealth Exchange connectivity	3.17
Governance	Data Use and Reciprocal Support Agreement	3.10
Use	Number of records exchanged using eHealth Exchange	3.00

**Table 2:** Findings of the interviews by descending order by category (relative frequency [RF] ≥3.00).

Category		RF	
**eHealth Exchange concerns or challenges**			
	Interoperability		6.17
	Level of implementation		4.60
	Increase statewide and regional health information exchange to eHealth Exchange connectivity		3.17
**Governance**			
	Data Use and Reciprocal Support Agreement		3.10
**Technical**			
	Technical standards		6.13
	Patient matching		5.80
	Data usability		4.37
	Data integrity		3.87
**Use**			
	Increase eHealth Exchange use		6.75
	eHealth Exchange participants		4.30
	Actual eHealth Exchange use time		3.30
	Number of records exchanged using eHealth Exchange		3.00
**Value**			
	Value in better care		6.77
	Value in avoiding duplication		4.93
	Value in lowering costs		4.47
	Social Security Administration Disability Determination		3.53

### Level of Implementation

Interview analysis suggested that few organizations are fully implemented, which would mean that they are performing queries, receiving and consuming the EHR usable information, and connected to federal partners. In terms of meaningful use, interviewees referred primarily to using eHealth Exchange as a vehicle for care transition summaries. Others described exchanging with federal partners as the level of implementation. When questioned further about implementation, many interviewees discussed other HIE networks used to exchange clinical data (eg, regional HIEs, vendor HIEs, specialized practice HIEs). Whether or not these HIEs were eHealth Exchange participants, a majority of the interviewees have implemented eHealth Exchange at the federal partner level for Social Security Administration (SSA) disability determination and/or the Veterans Health Administration (VHA).

### Increasing Statewide and Regional Health Information Exchange to eHealth Exchange Connectivity

Most interviewees thought that increasing statewide or regional HIE to eHealth Exchange connectivity would depend on a less cumbersome process to gain more traction. Regardless of processes that need streamlining (eg, testing and sign-in), interviewees suggested that statewide or regional HIEs should be the first level of connection to eHealth Exchange, then organizations and health systems should connect to their statewide or regional HIE. One interviewee stated, “I would say 90% to 100% of the time it [data from the statewide HIE] impacts the way that I deal with every single patient. There’s something on there that either changes the care that I would deliver... and because I’m aware of [a] clinical context, I’m just going to deal with that patient a little bit differently.” Such comments support the value of building a network of networks.

### Governance

Many interviewees thought that the Data Use and Reciprocal Support Agreement (DURSA), put in place by the Office of the National Coordinator for Health Information Technology (ONC) and carried forward by The Sequoia Project, was comprehensive and saved them significant legal counsel expenses (RF=3.10) to ensure that best practices, legislative regulations, and common sense are employed. Regional or statewide HIEs reported spending very few resources on DURSA review, which may be partially due to previous familiarity with the agreement. Overall, many interviewees said that there is a certain level of understanding and confidence that “we are all playing by the same rules.”

### Technical Standards

The technical issues most often expressed by interviewees were technical standards (RF=6.13), patient matching (RF=5.80), data usability (RF=4.37), and data integrity (RF=3.87). Importantly, no one mentioned technology as a barrier but rather raised selective technical areas that can be viewed as a natural consequence of the growth process.

Several interviewees commented about forward and backward compatibility between the 2010, 2011, and 2014 specifications. To provide some context around these comments, some organizations, such as SSA, support multiple versions, but this is not widespread. Organizations do not upgrade to the latest technical specifications in lockstep, so there will always be the need for forward and backward compatibility. For content standards, interviewees discussed the need for more granularity and more consistent interpretation of the standards. Two interviewees commented on the diversity of options for documenting data from the continuity of care document, although they acknowledged tighter specifications have resulted in improvements. Finally, some interviewees felt that vendors contribute to the lack of clarity with regard to standards—technical and content—and The Sequoia Project could help by setting universal standards and ensuring consistency in the interpretation and application of the standards between organizations and vendors.

### Patient Matching

Interviewees mentioned that accurate patient matching is a critical component to seamless health data exchange across eHealth Exchange. One interviewee stated the alternative very simply: “The fallout from inaccurate patient matching is too risky.” Interviewees linked patient matching to interoperability and data integrity saying that they may have made strides with patient matching for internal exchange within their organization, but more needs to be done specifically related to patient matching for external exchange across organizations (ie, eHealth Exchange). One interviewee suggested adoption of a nationwide patient matching strategy with standardized and vendor-agnostic patient demographic elements. Of those suggesting solutions, many mentioned a central patient list with a record locator and a unique health identifier (not the social security number).

### Data Usability

Two interviewees suggested that trust can be critical to how usable data are used. For example, if a clinician suspects that data may not be accurate for the patient (perhaps due to inaccurate patient matching), the data will be discounted and not perceived as useful. One of these interviewees when on to say that while the data may be accurate, there may be no need for those particular data. Importantly, interviewees with more HIE experience (regional or statewide) expressed that they feel the data they get *are* usable and helpful.

### Data Integrity

As one interviewee noted, “A fundamental and critical success factor for HIE is the ability to accurately link multiple records for the same patient across the disparate systems of the participating organizations.” Another interviewee added that this becomes an issue of patient safety when data are incorrectly merged, sometimes between the wrong patients, and the absence of accurate patient matching was seen by many interviewees as the root problem behind data integrity.

### Increasing eHealth Exchange Use

A majority of interviewee comments about use had to do with increasing eHealth Exchange use and usability (RF=6.75), understanding who is using eHealth Exchange (RF=4.30), the actual use time (RF=3.30), and the number of records exchanged using eHealth Exchange (RF=3.00).

Many interviewees felt that their organization’s prior experience with data exchange had resulted in increased use of eHealth Exchange; however, that use was primarily for SSA disability determination. Many felt that SSA use was high for 2 reasons: it did not require initiation from the user and there was concrete revenue tied to its use. Other than SSA disability determination, some interviewees noted that their organization did not have any set primary purpose for eHealth Exchange and thought that might be a contributing factor to low use.

One interviewee said that in their organization, it is possible that users are not even sure if they are using Epic or eHealth Exchange to query for records, as the query goes first to Epic and then to the eHealth Exchange without signaling the transition. This scenario, for this organization, runs about 8:1 Epic to Epic versus eHealth Exchange; another organization cited a 10:1 Epic to Epic versus eHealth Exchange ratio. In other words, Epic records are returned 8 or 10 times more frequently than eHealth Exchange records.

Some interviewees suggested that if insurance companies became eHealth Exchange participants, use would increase. Some organizations created their own connectivity with HIEs that existed prior to eHealth Exchange (or even NwHIN) and have not transitioned over. Other interviewees mentioned usability: “Asking for the data is one thing; getting usable information is quite another.”

### eHealth Exchange Participants

Interview data suggest that, outside of SSA and VHA, users are organization-to-organization rather than organization-to-HIE (statewide or regional). Many interviewees suggested that the lack of a provider directory contributes to low use. One interviewee stated, “Just knowing who your eHealth Exchange neighbors are might increase the propensity to initiate an eHealth Exchange query.”

### Actual eHealth Exchange Use Time

The interviewees were from organizations that had been eHealth Exchange (or NwHIN) participants ranging from 1 to 7 years. However, findings suggest that the length of eHealth Exchange (or NwHIN) participation did not necessarily reflect the length of time that organizations were electronically exchanging health data. Those who have been using eHealth Exchange the longest (some starting as NwHIN participants) commented that a majority of their exchanges are with SSA.

### Number of Records Exchanged Using eHealth Exchange

Many interviewees commented that the number of records exchanged using eHealth Exchange would rapidly increase if legislation made querying records a standard of care. However, these interviewees were quick to point out that doing so should not limit queries to only eHealth Exchange but from any HIE, including vendor systems such as Epic. Another issue brought up by several interviewees was enforcing data contribution: if an organization is an eHealth Exchange participant, they need to contribute data. Depending on the organization and regulations for sensitive data, this may be more complicated than it sounds.

In terms of actual records exchanged across eHealth Exchange, Table 3 lists in ascending order the average records transacted each month as reported by the interviewee.

### Value

While responses varied, it was apparent that all interviewees perceived value in being an eHealth Exchange participant. However, when queried for concrete value statements, interviewees mostly pointed to revenue generated from SSA participation. Most interviewees expressed that the primary perceived value was located in better care (RF=6.77), but others cited avoiding duplication of services (eg, lab, radiology) (RF=4.93). Again, although mostly anecdotal evidence, many mentioned lower costs of care as one of the value factors (RF=4.47). SSA disability determination (RF=3.53) was the only factor mentioned with a value that interviewees felt they could quantify for their organizations. It is critical to note that many interviewees who have been conducting health information exchange through regional HIEs anecdotally report better care, duplication avoidance, etc. These interviewees draw from these known experiences and perceive that this same value can and will happen at a national level with eHealth Exchange.

### Value in Better Care

**Figure 1:**
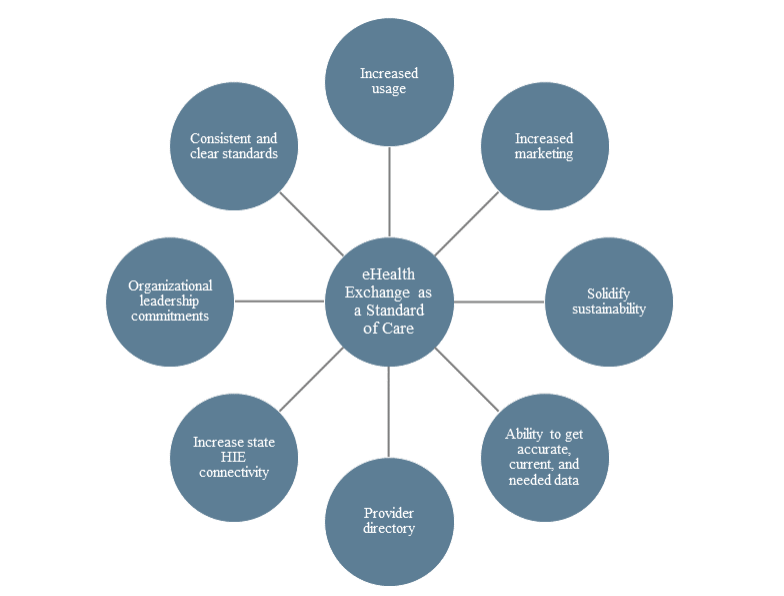
eHealth Exchange as a standard of care. HIE: health information exchange.

Several interviewees commented that although they think use of eHealth Exchange will result in better care, “its use must become the standard of care.” A network diagram constructed from interview data shows linkages to developing eHealth Exchange use as a standard of care. As shown in Figure 1, interviewees identified 8 core contributors to making eHealth Exchange a standard of care:

- Increased use
- Increased marketing
- Solidified sustainability
- Ability to get accurate, current, and needed data
- Provider directory
- Increased statewide HIE connectivity
- Organizational leadership commitments
- Consistent and clear standards

### Value in Avoiding Duplication of Services

Several interviewees commented that in order to avoid duplication of services, eHealth Exchange must get the patient matching right. One interviewee suggested that avoiding duplication of services could actually be motivated through a bottom-up approach with “the patient say[ing] that they just had that test, can you please check eHealth Exchange?”

### Value in Lowering Costs

Interviewees discussed the perceived value of eHealth Exchange in lowering overall costs of health care, and 1 interviewee commented, “While eHealth Exchange can play a strong role in lowering health care costs, we may not be able to attach causality to eHealth Exchange for a while.”

### Social Security Administration Disability Determination

Many interviewees commented that even though they are not seeing actual quantifiable value in terms of clinical outcomes, “Being an eHealth Exchange participant is the right thing to do for medicine.” One interviewee commented: “Revenue is not directly tied to why we’re part of the eHealth Exchange. We view participation with the eHealth Exchange as it’s just a part of who we are, and what we want to do, and how we promote interoperability in the country. I have to say I have never been in a meeting where we’d say, ‘Look, we’re making this amount of money from the SSA.’”

SSA disability determination was the only quantifiable value factor mentioned by interviewees, and for many, the primary motivation for their organization’s eHealth Exchange participation. One interviewee summed up the comments of many: “Credit to SSA for figuring out that [eHealth Exchange] was possible and then figuring out how to do it so it is of value.” To provide some context, uncompensated care cost recovery is directly linked to SSA disability determination; if an SSA beneficiary is approved for Social Security Disability Insurance (SSDI), they are more likely to pay the hospital bill and seek medical care before using expensive emergency care [4]. Most interviewees estimated the cost for eHealth Exchange onboarding $100,000 to $400,000 and very dependent of the existence of previous HIE participation.

**Table 3:** Monthly eHealth Exchange transactions.

Type of organization	Region	Average records per month^a^
Health care system	Southwest	10
Hospital	Southwest	667
State health information exchange	Midwest	1400
Veterans Health Administration	Federal	2000
Regional health information exchange	South	4000
Social Security Administration	Federal	25,657^b^

^a^These are estimates given by interviewees and represent both inbound and outbound transactions.

^b^Author’s analysis from Social Security Administration–provided data.

However, once onboarded, SSA participants estimated that the revenue generated from SSA queries largely offsets eHealth Exchange participation costs.

### Current and Perceived Value Factors

**Figure 2:**
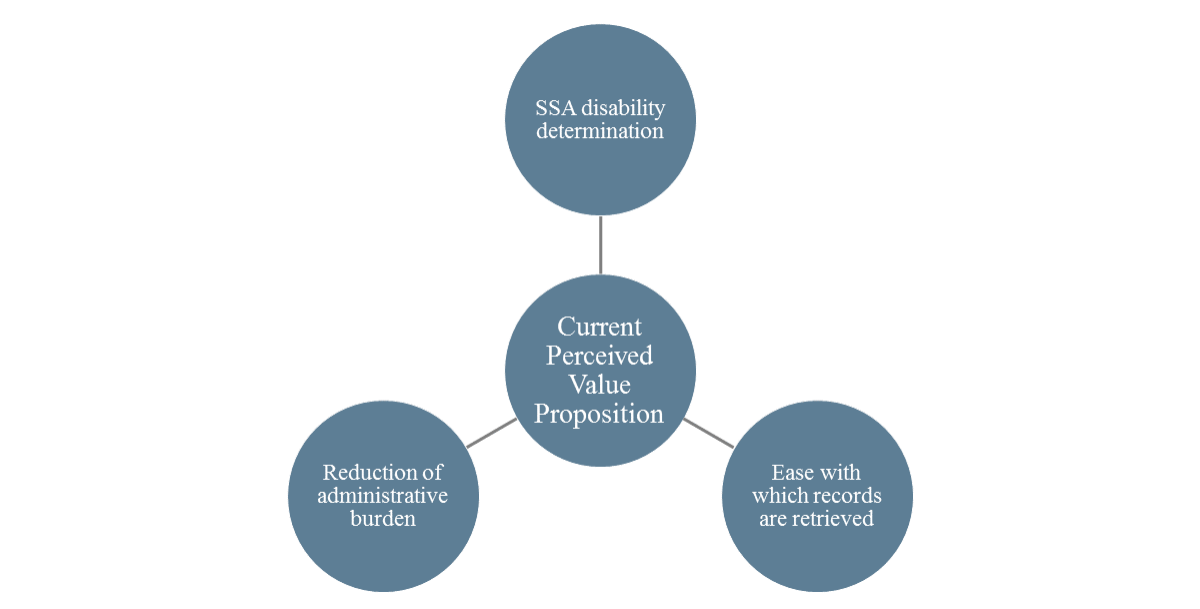
Current perceived value proposition. SSA: Social Security Administration.

**Figure 3:**
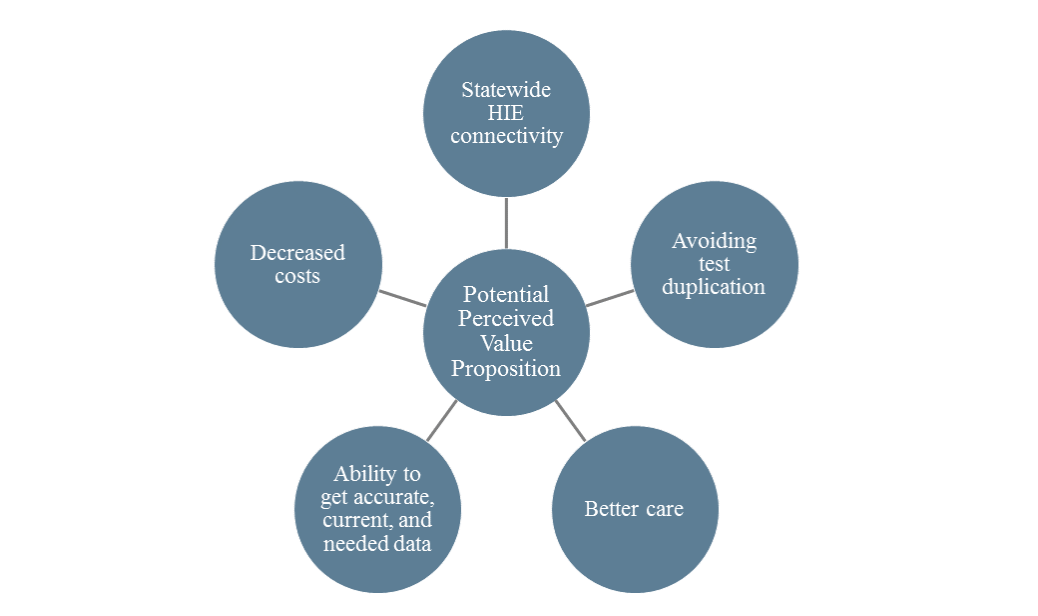
Potential perceived value proposition. HIE: health information exchange.

The interview data revealed linkages to current and future perceived value as shown in Figures 2 and 3, respectively. As shown in Figure 2, interviewees identified 3 core current perceived value factors:

- SSA disability determination (revenue and uncompensated care cost recovery)—this is the only value factor to have been quantified
- Ease with which records are retrieved
- Reduction of administrative burden for staff needing to request records

In terms of the potential perceived value—what interviewees expect will happen as eHealth Exchange evolves and has more participants—Figure 3 shows 5 core items:

- Statewide HIE connectivity
- Avoiding test duplication
- Better care
- Ability to get accurate, current, and needed data
- Decreased costs

### Health Information Exchange Model Development

**Figure 4:**
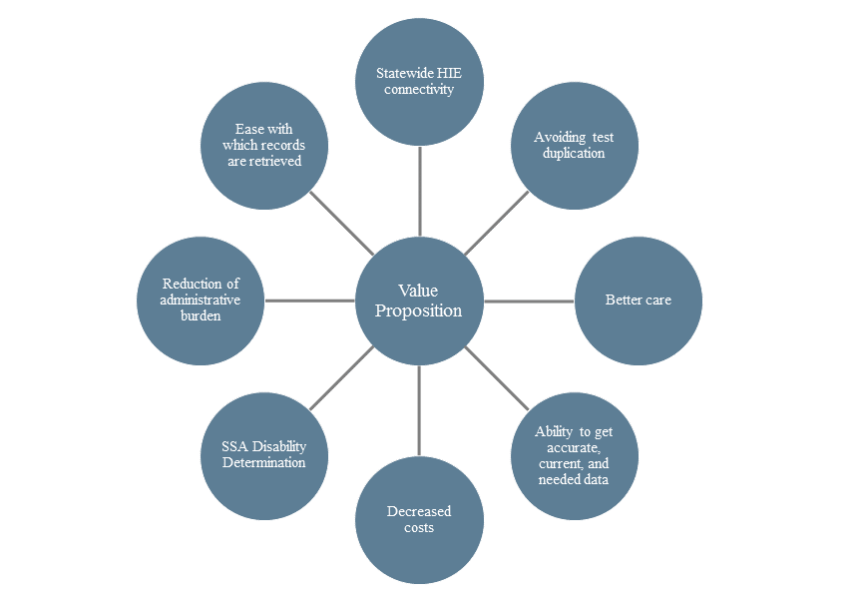
Model development for health information exchange value and sustainability. HIE: health information exchange; SSA: Social Security Administration.

The aforementioned factors that contribute to the current and potential perceived value provide the basis for model development for health information exchange value and sustainability (Figure 4). The next step is statistical testing of this model to understand the contribution of each factor in terms of dependent and mediating factors relative to value and sustainability.

## Discussion

### Principal Findings

Despite attention from policymakers and industry professionals, interoperability—the seamless exchange of health information among organizations for total patient care—remains elusive. Additionally, sharing health information across state lines is complicated by differing consent models (opt-in vs opt-out). As such, eHealth Exchange participants are not waiting for a perfect network but rather are willing to participate in what they can achieve now and readily avail themselves to the advantages of group forming networks (Reed’s Law): group-to-group connections where each single connection creates a much larger network for health information exchange. This differs from those who are withholding eHealth Exchange onboarding until a tipping point of value has been achieved. It is likely that those who are current participants will see much earlier and much greater return on their investment and, more importantly, will be able to quantify elements of the Triple Aim. In ways that may not yet be apparent, such positioning could offer a strategic advantage to providing health care to patients from anywhere in the United States. By contrast, the primary findings from this study that factor into value could also impact or influence future value, especially if there is no further maturity of eHealth Exchange. It is also important to understand that even with complete interoperability, there could still be a lack of complete medical information, leading to a lack of trust in any of the information.

### Group Forming Networks

eHealth Exchange has demonstrated usefulness in facilitating the development of group forming networks, as this enables health care providers to connect not only to each other but also to federal entities that have a vested interest in improving care quality (eg, VHA).

### Value and Sustainability

There are several opportunities to enhance the value and thus the sustainability of eHealth Exchange. The first is to improve consumer engagement by educating patients on the value of health data exchange through an HIE. Doing so will create a culture of patients who expect and demand health data sharing as a standard of care. Additionally, compiling a national provider directory or a similar mechanism for eHealth Exchange participants will enable care providers to readily identify with whom they can exchange information.

In a similar vein, interviewees expressed that increasing awareness and usefulness of eHealth Exchange would prove beneficial to increasing value and sustainability. While there is much anecdotal discussion around what participants feel is working, very little of it has been formalized with studies. Additionally, interviewees commented on the need for increased marketing endeavors. Exchangeability for current eHealth Exchange participants can be increased by focusing on onboarding statewide HIEs and organizations in states neighboring current participants.

Another method to increase the value of eHealth Exchange is to quantify use cost and clinical outcomes through studies on well-established use cases for eHealth Exchange. Other benefits worth considering include decreased duplication for laboratory or radiology services and reduced admission rates from emergency department visits.

Ensuring data integrity and patient matching are priorities, with standardized processes to ensure overall data integrity and thus confidence in the information presented at the point of care. It is recommended that The Sequoia Project combine the findings from this study with public comments received from the recently released report entitled “A Framework for Cross-Organizational Patient Identity Management” [13].

The need to advance interoperability was mentioned by nearly every interviewee. It is recommended to use policy and funding levers to create a business imperative and clinical demand for interoperability. This may require greater involvement of the federal government to align economic incentives, including but not limited to a stronger commitment from the Centers for Medicare and Medicaid Services, which could take a multitude of forms but should start with something manageable, actionable, and measurable such as requiring all emergency department visits with an ambulatory sensitive condition diagnosis to have an external HIE query.

### Model Development

While the above enhancement opportunities provide guidance to eHealth Exchange, parallel discovery is needed in understanding the strength of the constructs that contribute to the model suggested in Figure 4. This study combines current and potential perceived value to provide the basis for early model development for health information exchange value and sustainability. This model then needs to be statistically tested to determine the strength of each of the constructs and to what degree they are mediating or contributing factors.

### Limitations

Limitations of this study include purposeful study participant recruitment of current eHealth Exchange participants who were involved in the decision-making process to onboard to eHealth Exchange. Future research would benefit from including end users. Additionally, several characteristics of eHealth Exchange are not applicable to health information exchange as it occurs broadly through other types of HIEs, such as those that are vendor supported. As such, the findings may or may not be applicable to health information exchange broadly.

### Conclusion

Organizations interested in sharing electronic health information are not waiting for perfection in the HIE infrastructure (eHealth Exchange, state or local HIE) to engage in that sharing, but rather they are identifying particular use cases to demonstrate value. They are also relying on the advantages of group forming networks to increase the value of various use cases. Engagement of the consumer is emerging as a critical component in the value equation for health data exchange. With consumers having increased awareness of health data exchange, they stand to drive the future for health data exchange becoming a standard of care. However, absent data integrity and interoperability, the value equation will continue to be built on individually identified use case factors. This study looked at value from a variety of factors that contribute to value and sustainability. Future research will test this model to better understand the strength of each factor.

## Appendix

Multimedia Appendix 1 Interview questions.

## Abbreviations

DURSA: Data Use and Reciprocal Support Agreement
EHR: electronic health record
HIE: health information exchange (a single organization or group of organizations facilitating the act of electronic health information exchange)
HIMSS: Healthcare Information and Management Systems Society
HITECH: Health Information Technology for Economic and Clinical Health
NwHIN: Nationwide Health Information Network (now eHealth Exchange)
ONC: Office of the National Coordinator for Health Information Technology
RF: relative frequency
SSA: Social Security Administration
SSDI: Social Security Disability Insurance
VHA: Veterans Health Administration
