# Supplementary material for: An Early Model for Value and Sustainability in Health Information Exchanges: Qualitative Study
Source: JMIR Med Inform. 2018 Apr 30;6(2):e29. doi: 10.2196/medinform.9299 (PMC5952116; doi:10.2196/medinform.9299)
Supplement: Multimedia Appendix 1 [file medinform_v6i2e29_app1.pdf]

Multimedia Appendix 1

Below is the full interview protocol for questions related to this publication.

Technical

1. What were the initial technical processes needed (vs. available) when you decided to join eHealth Exchange?

2. What were your initial thoughts regarding data availability, accessibility, and usability? What are they now?

3. What technical advances will need to happen for more organizations to join eHealth Exchange?

4. What health IT related technical standards need to change and why?

5. What technical issues need to be solved for to impact sustainability?

6. What are the opportunities to be considered in HIE maturity?

Organizational

1. What was the initial perceived value of electronic data sharing to the goals of your organization? What are they now?

2. What organizational challenges emerged and how were they addressed? Any remaining?

3. What are the current reasons for maintaining your participation in eHealth Exchange?

4. To what degree did you think that data is or can be used to improve care delivery, reduce costs, etc. And now?

5. How do you think the motivations for and value of eHealth Exchange participation will change in the future? Overall and from your organizations perspective.

Governance

1. What public policies need to happen for hIE to be a standard of care?

2. What governance structures do you see as vital for sustainability or growth of electronic data sharing?
